# Effect of Distance to Trauma Centre, Trauma Centre Level, and Trauma Centre Region on Fatal Injuries among Motorcyclists in Taiwan

**DOI:** 10.3390/ijerph18062998

**Published:** 2021-03-15

**Authors:** Bayu Satria Wiratama, Ping-Ling Chen, Chung-Jen Chao, Ming-Heng Wang, Wafaa Saleh, Hui-An Lin, Chih-Wei Pai

**Affiliations:** 1 Graduate Institute of Injury Prevention and Control, College of Public Health, Taipei Medical University, Taipei City 110, Taiwan; bayu.satria@ugm.ac.id (B.S.W.); plchen@tmu.edu.tw (P.-L.C.); sevenoking219@gmail.com (H.-A.L.); 2 Department of Epidemiology, Biostatistics, and Population Health, Faculty of Medicine, Public Health and Nursing, Universitas Gadjah Mada, Yogyakarta City 55281, Indonesia; 3 Department of Traffic Science, Central Police University, Kueishan District, Taoyuan City 33304, Taiwan; una051@mail.cpu.edu.tw; 4 Department of Traffic Management, Taiwan Police College, Taipei City 110, Taiwan; wang.mingheng@gmail.com; 5 Transport Research Institute, Edinburgh Napier University, Edinburgh EH11 4DY, UK; w.saleh@napier.ac.uk; 6 Department of Engineering, Princess Nora Bint Abdul Rahman University, Riyadh 11564, Saudi Arabia; 7 Department of Emergency Medicine, Taipei Medical University Hospital, Taipei City 110, Taiwan

**Keywords:** fatal injury, motorcyclist, motorist, traffic crashes, trauma centre

## Abstract

Background: Studies have suggested that trauma centre-related risk factors, such as distance to the nearest trauma hospital, are strong predictors of fatal injuries among motorists. Few studies have used a national dataset to study the effect of trauma centre-related risk factors on fatal injuries among motorists and motorcyclists in a country where traffic is dominated by motorcycles. This study investigated the effect of distance from the nearest trauma hospital on fatal injuries from two-vehicle crashes in Taiwan from 2017 to 2019. Methods: A crash dataset and hospital location dataset were combined. The crash dataset was extracted from the National Taiwan Traffic Crash Dataset from 1 January 2017 through 31 December 2019. The primary exposure in this study was distance to the nearest trauma hospital. This study performed a multiple logistic regression to calculate the adjusted odds ratios (AORs) for fatal injuries. Results: The multivariate logistic regression models indicated that motorcyclists involved in crashes located ≥5 km from the nearest trauma hospital and in Eastern Taiwan were approximately five times more likely to sustain fatal injuries (AOR = 5.26; 95% CI: 3.69–7.49). Conclusions: Distance to, level of, and region of the nearest trauma centre are critical risk factors for fatal injuries among motorcyclists but not motorists. To reduce the mortality rate of trauma cases among motorcyclists, interventions should focus on improving access to trauma hospitals.

## 1. Introduction

Research has highlighted that trauma systems can reduce mortality risk among patients with traumatic injuries [1,2]. Although the focal point of trauma systems are trauma centres, prehospital factors, including prehospital transport time and care, crucially affect survival rates [3,4,5]. Studies have indicated that people living in rural and low-income areas and those belonging to minority communities have difficulties accessing trauma care, suggesting that disparities in health care systems, built environments, and social environments cause differences in health outcomes [6,7,8].

Research on prehospital factors, such as hospital distance and prehospital time, has revealed several risk factors associated with increased fatality rates in cases of trauma. In a study examining only major trauma, Feero et al. [3] concluded that shorter prehospital time had a positive effect on survival. By contrast, a longer total prehospital time is associated with in-hospital trauma mortality [5]. Trauma centre distance is also associated with an increased risk of death after prehospital time and other risk factors are controlled [9]. By using Fatality Analysis Reporting System data from 2010 to 2012, Hu, Dong, and Huang [10] reported an association between trauma centre distance and fatal injuries sustained in motor vehicle crashes. A US study by Durkin et al [11]. concluded that fatal injuries were more likely to occur from crashes located >30 miles from the nearest level-I or level-II trauma centres in Wisconsin. Gonzalez et al. [12] used police crash reports and emergency medical service (EMS) patient care reports to demonstrate that increased EMS response time and time on scene were associated with higher mortality rates among patients in motor vehicle crashes. By using National Emergency Medical Services Information System data, Byrne et al. [13] also concluded that longer EMS response times were associated with an increased mortality rate among people in motor vehicle crashes.

Studies have concluded that factors related to trauma systems, such as greater distance to trauma centre, lower density of the trauma centre, and ground ambulance compared with helicopter availability, are positively associated with higher mortality rates among trauma patients. In the United States, the implementation of an organised trauma system appeared to significantly affect motor vehicle crash mortality [14]. Byrne et al. [13] indicated that among 2268 US counties, the presence of a level-I or level-II trauma centre contributed to lower mortality rates for those in motor vehicle crashes. Compared with patients transported by helicopter, those transferred by ground ambulance had higher mortality rates [15], which was primarily due to longer prehospital times. Demetriades et al. [16] reported that patients transferred to level-I trauma centres had a lower risk of sustaining fatal injuries than did those transferred to trauma centres with level-II or lower status. Another study conducted in the United States demonstrated that states with clustered trauma centres had lower median injury fatality rates than did states with dispersed trauma centres [17].

Other risk factors are associated with fatal injury among trauma patients, such as economic status, area of residence, and individual characteristics. Rural counties with low population densities are associated with a higher rate of mortality from motor vehicle crashes [13]. Sex, age, and injury type are also associated with mortality rates among trauma patients [9,15]. Several studies have also suggested that rural areas, low-income areas, and minority communities are associated with inaccessible trauma care [7,8], which may increase mortality rates.

Relevant studies have demonstrated that increased hospital distance is associated with a higher risk of fatal injuries [11,12,18]. Although these studies have used only city-level data, few risk factors have been controlled as confounding variables. Currently, few studies have adopted national-level data to investigate the effect of hospital distance on fatal injuries, particularly in countries, such as Taiwan, where traffic is dominated by motorcycles. The effect of hospital distance on fatal injuries may be more pronounced among motorcyclists than motorists. This study investigated the effect of distance to the nearest trauma centre on fatal injuries among motorcyclists involved on 2-vehicle crashes in Taiwan by using the National Taiwan Traffic Crash Dataset from 2017 to 2019. This study also aims to compare the effect of distance to the nearest trauma centre between motorcyclists and motorist.

## 2. Materials and Methods

### 2.1. Data Source

This is an observational study using national datasets. The current study combined two datasets, namely a crash dataset and hospital location dataset. The crash dataset was extracted from the National Taiwan Traffic Crash Dataset from 1 January 2017 through 31 December 2019. This dataset contains every traffic crash reported to the police in Taiwan and includes three categories of crash data, namely crash, vehicle, and victim files. Data were recorded by experienced investigators. The crash files contain data on the characteristics of the crash, such as the date and time of the crash, weather conditions, road type, coordinates of the crash location, and crash type. Data on vehicle characteristics, such as the first point of impact, vehicle type, and vehicle manoeuvres, are included in the vehicle file. Data on victim characteristics, such as sex, age, injury severity, license status, blood alcohol concentration (BAC), and protective device use (seatbelt or helmet) are also recorded in the victim file. Injury-related data were obtained from hospital records by police officers. According to Taiwanese law, hospitals must regularly report cases related to traffic crashes to the police. Two spatial variables were used in this study, namely crash location and trauma centre location. Crash location was obtained from the crash dataset, specifically the crash file section, and was defined as the latitude and longitude of the crash site obtained from the police. The trauma centre location data set consisted of data on trauma centres in Taiwan, such as the names of trauma centres, trauma centre levels (I, II, or III), and trauma centre addresses. These data were acquired from the Ministry of Health and Welfare of Taiwan [19]. Trauma centre addresses were batch geocoded into longitudes and latitudes by using the geocode function of QGIS. This study was approved by Taipei Medical University institutional review board (IRB) with IRB number N202008027.

This study analysed data related with 2-vehicle crashes in Taiwan. Of a total of 550,995 cases of 2-vehicle crashes, those with missing data (*n* = 2802), such as license information and data on the use of protective equipment, were excluded in this study. This study used the complete case analysis approach as proposed by Kang [20]. No significant differences were detected between cases with and without missing data (*p* > 0.05). Based on this result, this study concluded that the occurrence of missing data on our dataset was random. Therefore, this study excluded cases with missing data from our analysis. The final data set consisted of 550,995 cases of 2-vehicle crashes.

### 2.2. Variable Definitions

The main outcome variable was injury severity, which was categorised as fatal and nonfatal injury. Fatal injury was defined as motorists or motorcyclists sustaining fatal injuries within 24 h after a traffic crash and nonfatal injury as motorists or motorcyclists sustaining any type of injury other than a fatal injury who survived for more than 24 h after the crash. Based on past studies, several independent variables were included into this study as follows: sex [21,22], age [21,22,23,24], protective device use [21,22], BAC [21,22], license status [21,22,23,24], vehicle type [21,22,23,24], weather condition [21,22], road light condition [21,22], road speed limit [21,22], crash type [22], distance to the nearest trauma centre [9,13,25], region of trauma centre [13], and trauma centre level [13]. Sex and age data were collected for both drivers and their crash partners. Age was categorised into the following four groups: <18 years, 18 to 40 years, 41 to 64 years, and ≥65 years. Drivers younger than 18 years were classified as illegal drivers because of their inability to obtain a valid driver’s license in accordance with Taiwanese law. Drivers aged ≥65 years were considered to be elderly and have an increased risk of fatal injuries. Those aged 18 to 64 years were divided into two nearly equal age groups of 18 to 40 and 41 to 64-year-old drivers.

Data on driver factors, namely protective device use, BAC, and license status, were collected. Protective device use was defined as using or not using protective equipment such as seatbelts or helmets during road traffic crashes. BAC was classified as positive when the driver’s BAC was ≥0.03% and negative when it was <0.03% [21,22]. This classification was used because driving is illegal in Taiwan at a BAC ≥ 0.03% and results in a fine. For the license variable, drivers with a valid license were coded as “yes”, and those without a valid license were coded as “no”.

This study collected data on vehicle type for both the driver and the driver of the other vehicle (hereafter “crash partner”) in a crash. Vehicles were classified into four types: motorcycles, heavy vehicles (e.g., trucks or buses), taxis, and personal cars or vans. Road environmental factors, namely weather conditions (adverse or fine weather), road light conditions (night-time or daytime), and road speed limit (≥50 km/h or <50 km/h), were analysed. Road light conditions were defined as “daytime” if crashes occurred between sunrise and sunset and “night-time” if crashes occurred between sunset and sunrise [22,26]. Data on sunrise and sunset times in Taiwan were obtained from the National Oceanic Earth System Research Laboratory and Atmospheric Administration database (accessed 2 February 2020). Crash type was classified into 5 of the following types in accordance with a previous study (Wiratama et al. [22]): head-on, rear-end, sideswipe, angle, and other.

The distance between the crash location and the nearest trauma centre was measured according to the Euclidean (straight-line) distance. This study used a geographic information system to perform Euclidean distance analysis and nearest neighbour analysis to identify the nearest trauma centre to each crash location. Cudnik et al. [27] demonstrated a high correlation between actual road network distance and Euclidean distance (*r* = 0.97). Therefore, Euclidean distance was used as a surrogate measure of actual road network distance. This study used distance matrix function in QGIS [28] under the vector analysis command group. This study uses only one nearest target point to get distance to the nearest trauma centre from crash locations. Distance to the nearest trauma centre was classified into two categories: ≥5 km and <5 km. The cut-off of 5 km was selected on the basis of two reasons: first, several analyses were conducted using different cut-off points and this study found that 5 km had the highest risk of fatal injuries (please refer to Table 1); second, the cut-off point was also confirmed from research by Hu et al. [10], which demonstrated that distances of >5 km to a hospital are associated with a higher mortality rate than distances of <5 km. The other two variables for trauma systems in the present study were trauma centre level (I, II, or III) and hospital region (East, South, Central, or North Taiwan). Level I is considered the highest level for trauma centres in Taiwan.

### 2.3. Statistical Analysis

This study presents the distribution of injury severity (i.e., fatal and nonfatal injury) by all risk factors with absolute numerical values and percentages. For statistical analysis, we conducted bivariate and multivariate analysis stratified by motorcyclists and motorists. A stratified analysis was conducted to compare the effect of distance to the nearest trauma centre on fatal injuries between motorcyclists and other motorists. In the bivariate analysis, a simple logistic regression was used to examine the association between all risk factors and fatal injuries. In accordance with previous research, *p*-value < 0.2 was the cut-off point for including risk factors in the multivariate analysis [21,22]. A multiple logistic regression analysis was performed to calculate the adjusted odds ratios (AORs) for fatal injuries. This study used Strengthening the Reporting of Observational Studies in Epidemiology guidelines [29] for reporting an interaction effect between hospital distance and hospital region. The interaction variables were classified into the following eight groups: (1) <5 km and northern region (as reference); (2) <5 km and central region; (3) <5 km and southern region; (4) <5 km and eastern region; (5) ≥5 km and northern region; (6) ≥5 km and central region; (7) ≥5 km and southern region; and (8) ≥5 km and eastern region. This study calculated Cramer’s *V* and used a chi square independent test to assess multicollinearity among risk factors. This study used 95% CIs and α = 0.05. STATA version 15 (StataCorp LLC, College Station, TX, USA)was used to analyse data in this study [30].

## 3. Results

### 3.1. Characteristics of Crashes

Table 2 presents the characteristics of crashes in this study. Between 2017 and 2019, there were in total 550,995 casualties, of which 1570 (0.28%) involved fatal injuries and 549,425 (99.72%) involved nonfatal injuries. More fatal injuries occurred in crashes located ≥5 km from the nearest trauma centre (0.73%) than in crashes located <5 km from the nearest trauma centre (0.24%). Fatal injuries were more prevalent among crashes that occurred near level-III trauma centres (0.36%), followed by level-II (0.28%) and level-I (0.20%) trauma centres. Motor vehicle crashes in East Taiwan resulted in the largest proportion of fatal injuries (0.53%) relative to crashes in other regions.

### 3.2. Risk Factors for Fatal Injuries among Motorcyclists

Table 3 presents the results of the simple logistic regression models for motorcyclist casualties. Motorcyclists in crashes located ≥5 km from the nearest trauma centre had a 3.2-times higher risk (OR = 3.20; 95% CI = 2.85–3.60) of sustaining fatal injuries than did those in crashes located <5 km away from a trauma centre. Motorcycle crashes that occurred near level-III and level-II trauma centres had a 77% (OR = 1.77; 95% CI = 1.52–2.05) and 40% (OR = 1.40; 95% CI: 1.22–1.62) higher probability of involving fatal injuries, respectively, relative to motorcycle crashes that occurred near level-I trauma centres. Motorcyclists involved in crashes in East Taiwan were 2.46-times more likely to sustain fatal injuries (OR = 2.46; 95% CI: 1.91–3.16) than were those involved in crashes in North Taiwan.

### 3.3. Risk Factors for Fatal Injuries among Motorists

The risk factors of fatal injuries among motorists are reported in Table 3. Motorists in crashes located ≥5 km from the nearest trauma centre were 1.95-times more likely (OR = 1.95; 95% CI: 1.19–3.17) to sustain fatal injuries than were those in crashes located <5 km from the nearest trauma centre. Motorists involved in crashes near level-III trauma centres had a 2.47-times higher risk (OR = 2.47; 95% CI: 1.22–5.01) of sustaining fatal injuries than did those involved in crashes near level-I trauma centres. The effect of level-II trauma centres on fatal injuries was nonsignificant.

### 3.4. Multivariate Results of Risk Factors for Fatal Injuries among Motorcyclists

Table 4 lists the results of the multiple logistic regression for motorcyclists. Distance to the nearest hospital was a significant risk factor for fatal injuries; the odds of sustaining fatal injuries was approximately 2-times higher (AOR = 1.98; 95% CI: 1.75–2.24) for crashes located ≥5 km from the nearest trauma centre relative to crashes located <5 km from the nearest trauma centre. After other risk factors were controlled, trauma centre level remained a significant risk factor for fatal injuries among motorcyclists, with an approximately 30% (AOR = 1.30; 95% CI: 1.11–1.51) and 19% (AOR = 1.19; 95% CI: 1.03–1.37) higher likelihood of crashes occurring near level-III and level-II trauma centres, respectively, relative to crashes occurring near level-I trauma centres. Crashes in East Taiwan were 76% more likely (AOR = 1.76; 95% CI: 1.35–2.29) to result in fatal injuries than were those in North Taiwan. Motorcyclists with a positive BAC had a 4.1-times higher likelihood of sustaining fatal injuries (AOR = 4.10; 95% CI: 3.38–4.98) relative to those with a negative BAC. Motorcyclists involved in head-on crashes were 65% (AOR = 1.65; 95% CI: 1.40–1.95) more likely to sustain fatal injuries than were those involved in angle crashes. The multicollinearity test revealed no multicollinearity between independent variables in this study.

### 3.5. Multivariate Results of Risk Factors for Fatal Injuries among Motorists

Risk factors for fatal injuries among motorists as revealed in the multiple logistic regression are listed in Table 4. Crashes ≥ 5 km from the nearest trauma centres, trauma centre level, and region were all nonsignificant risk factors after other risk factors had been controlled. Motorists who did not use seatbelts had an approximately 9-times higher likelihood (AOR = 8.67; 95% CI: 5.31–14.14) of sustaining fatal injuries than did those who did use seatbelts. Motorists involved in head-on crashes were 12-times more likely (AOR = 12.01; 95% CI: 4.82–29.88) to sustain fatal injuries than were those involved in angle crashes.

### 3.6. Interaction Effects of Distance to the Nearest Trauma Centre and Trauma Centre Region

One specific interaction term, namely for the interaction between the distance to the nearest trauma centre and region, was incorporated into the multiple logistic regression models for motorcyclists and motorists (Table 5). The interaction effect was more pronounced among motorcyclists than motorists. Motorcyclists involved in crashes located ≥5 km from the nearest trauma centre and in East Taiwan had an approximately 5-times higher likelihood (AOR = 5.26; 95% CI: 3.69–7.49) of sustaining fatal injuries than did those involved in crashes located <5 km from a trauma centre and in North Taiwan. In the model for motorists, no significant interactions between distance to the nearest trauma centre and trauma centre region were detected.

## 4. Discussion

To our knowledge, this is the first study to use nationwide data and report an association between geography-related risk factors and fatal injuries in 2-vehicle crashes after demographic and crash-related variables are controlled. Previous studies have analysed trauma centre distance and fatal injuries by using only state- or city-level data and were unable to account for crash-related variables [9,10,11,13]. Because nationwide data with crash-related variables were used, the results obtained in this study may be more representative than those of previous studies relying solely on state- or city-level data.

Notably, this study demonstrated that a distance of ≥5 km to the nearest trauma centre was associated with a higher risk of fatal injuries among motorcyclists after other risk factors were controlled. Previous studies on the occupants of other motor vehicles have similarly concluded that a greater distance to the nearest trauma centre results in a higher probability of fatal injuries [9,10,11,18]. This increased risk of fatal injuries may be attributable to longer delays to in-hospital care and an increased mortality rate, as suggested by Brown et al. [18]. Evidence in the literature has indicated that prehospital time, including response and transport time, is positively associated with distance to a trauma centre [31]. Another study on patients with stroke demonstrated a similar result that transportation time is positively correlated with distance to the hospital [32].

In this study, we only had data on distance to the nearest trauma centre. Several other factors related to trauma systems were not considered in this study, such as triage status and prehospital time. Trauma centre distance may be associated with geographic variation in triage and EMS protocols or other trauma system-related factors. Furthermore, data on trauma system protocols for triage and transfer to another trauma centre were unavailable in this study. However, this study reports a crucial finding that the distance to the nearest trauma centre is associated with injury severity. Nevertheless, such a finding should be interpreted with caution because other trauma system-related factors were not considered. Future studies are warranted to examine prehospital events, prehospital time, triage protocols, and EMS system characteristics and their relationship with the distance to trauma centres.

The more pronounced effect of distance to the nearest hospital on fatal injury among motorcyclists may be due to differences in injury severity between motorcyclists and motorists. Previous studies have concluded that motorcyclists are more likely to sustain severe injuries than are motorists [33,34,35]. Lule, Ssebuufu, and Okedi [36] also reported that longer prehospital time was associated with an increased risk of severe injury related to femoral fractures among motorcyclists. On the basis of these findings, improved access to trauma centres is particularly crucial for motorcyclists.

In accordance with previous studies [9,13,37], our findings indicate that trauma centre level is a significant predictor of fatal injuries among motorcyclists. Such an effect may be attributable to the tendency of higher-level trauma centres to have more resources, such as diverse trauma specialists (e.g., neurosurgeons, orthopaedic surgeons, anaesthesiologists, and general surgeons), trained trauma staff, and trauma-related medical equipment. The study conducted by Kim et al. [38] demonstrated that level-I trauma centres are more likely to have in-house trauma-related physicians than level-II trauma centres are. The availability of trauma-related physicians was negatively associated with injury mortality rates in a study by Melton et al. [39].

Traffic crashes in East Taiwan, where hospitals are more dispersed that those in North Taiwan are [19], were more likely to result in fatal injuries than were those in North Taiwan. This result is similar to that of Brown et al. [17], who demonstrated that regions with dispersed trauma centres are positively associated with an increased risk of fatal injuries among motor vehicle drivers. Another study by Yang et al. [40] supported this result that motor vehicle crashes in rural and mountainous areas have higher mortality rates that those in other areas. Such an effect may be due to differences in the geographic distributions of trauma centres and populations with access to prehospital care. Previous studies have suggested that prehospital time, including activation, response, on-scene, and transport time, is significantly greater in rural areas than in urban areas [31].

To our knowledge, this is the first study to report a significant interaction between distance to a trauma centre and region of crash. Motorcyclists involved in crashes located ≥5 km from the nearest trauma centre and in East Taiwan had a higher risk of fatal injuries than did those involved in crashes located <5 km from the nearest trauma centre and in North Taiwan. Such an effect is attributable to the lower access to trauma care among people living in rural areas than among those living in urban areas [7], which may result in an increased risk of fatal injuries as a result of motor vehicle crashes.

This study discovered that driving under the influence of alcohol is positively associated with fatal motor vehicle crashes, as supported by previous studies [21,22,41,42]. This finding is likely attributable to alcohol-related impairment in vision, hearing, cognition, and executive function [43,44,45], which may increase the risk of fatal injuries.

Evidence in the literature has indicated that head-on crashes are a significant risk factor of fatal injuries [21,23,46,47,48]. Consistent with these studies, this study concluded that motorists involved in a head-on crash had a higher risk of sustaining fatal injuries. Such an effect may be associated with the increased probability of speeding among drivers involved in head-on crashes [49,50]. A study conducted by Afukaar [51] reported that speeding was highly associated with fatal injuries because the high impact force exceeds human tolerance. Other studies have revealed that drivers involved in head-on crashes are more likely to sustain head, cervical, and chest injuries [52,53,54,55], which generally lead to unfavourable trauma outcomes.

This study has several intrinsic limitations. First, this study could not collect data on injuries (e.g., head or cervical injuries) apart from discrete injury severity (i.e., fatal injuries) because this study used only the National Taiwan Traffic Crash Dataset. Future studies can link the crash dataset with hospital-related data, such as data from the National Health Insurance Research Database. Second, several crucial variables could not be controlled in this study, such as prehospital time, time spent at the scene of a crash, transport time, distance to the nearest emergency medical team, and other geography-related risk factors. Further study is warranted to control or analyse other geography-related risk factors. Third, this study was unable to exclude those who died at the crash scene because of a data limitation; precise status of death is unavailable in the police database. This data limitation means that these results must be interpreted with caution because the presence of a trauma system and timely response does not affect victims who die on the scene of a crash. Fourth, only the geographical distance between crash locations and the nearest trauma centres could be estimated. Other relevant studies have also used geographic distance as a measure of distance to trauma centres [9,18]. Furthermore, road network data were unavailable in this study, and QGIS software was unable to calculate the travel distance between a crash scene and the nearest trauma centre. Therefore, this study was only able to calculate the geographical distances between crash locations and their nearest trauma centres. Future research is needed to compare the results of using geographic distance to the nearest trauma centre with those of using other measures, such as the distance to EMS stations, the travel distance, and other related spatial variables. Finally, only the Taiwanese national crash dataset was used in this study. Therefore, these results may be unique to Taiwan or other countries with similar characteristics. Studies using data from other jurisdictions should confirm the generalisability of this results to different settings.

## 5. Conclusions

This study demonstrated that distance to the nearest trauma centre is a risk factor for fatal injuries from vehicle crashes, especially among motorcyclists. Riders involved in crashes located ≥5 km from the nearest trauma centre had approximately double the risk of sustaining fatal injuries than did those in crashes located <5 km from the nearest trauma centre. Based on these findings, improved access to trauma centres is particularly crucial for motorcyclists. Future studies are warranted to examine prehospital events, prehospital time, triage protocols, EMS system characteristics and their relationship with distance to the trauma centres.

## Figures and Tables

**Table 1:** Analysis of different cut-off points for distance to the nearest trauma centre.

Distance to the Nearest Trauma Centre	OR	95% CI
1 km cut-off point ≥1 km <1 km	1.52	1.46–1.84
2 km cut-off point ≥2 km <2 km	1.67	1.52–1.95
3 km cut-off point ≥3 km <3 km	1.73	1.64–2.02
4 km cut-off point ≥4 km <4 km	1.78	1.65–2.10
5 km cut-off point ≥5 km <5 km	1.98	1.75–2.24
6 km cut-off point ≥6 km <6 km	1.92	1.80–2.40
7 km cut-off point ≥7 km <7 km	1.96	1.85–2.54

**Table 2:** Characteristics of casualties.

Characteristics	Driver’s Injury Severity		Total *n* (%)
	Fatal *n* (%)	Nonfatal *n* (%)	
Driver’s age			
≥65 years 41 to 64 years <18 years 18 to 40 years	554 (0.92)	59,792 (99.08)	60,346 (10.95)
	482 (0.31)	153,532 (99.69)	154,014 (27.95)
	32 (0.36)	8867 (99.64)	8899 (1.62)
	502 (0.15)	327,234 (99.85)	327,736 (59.48)
Crash partner driver’s age			
≥65 years 41 to 64 years <18 years 18 to 40 years	113 (0.25)	45,744 (99.75)	45,857 (8.32)
	690 (0.31)	221,416 (99.69)	222,106 (40.31)
	11 (0.24)	4549 (99.76)	4560 (0.83)
	756 (0.27)	277,716 (99.73)	278,472 (50.54)
Driver’s sex			
Male Female	1098 (0.36)	307,492 (99.64)	308,590 (56.01)
	472 (0.19)	241,933 (99.81)	242,405 (43.99)
Crash partner driver’s sex			
Male Female	1313 (0.35)	376,530 (99.65)	377,843 (68.57)
	257 (0.15)	172,895 (99.85)	173,152 (31.43)
Driver’s use of protective equipment			
Not using a seatbelt or helmet Using a seatbelt or helmet	339 (0.81)	41,386 (99.19)	41,725 (7.57)
	1231 (0.24)	508,039 (99.76)	509,270 (92.43)
Driver’s BAC			
Positive Negative	150 (2.03)	7222 (97.97)	7372 (1.34)
	1420 (0.26)	542,203 (99.74)	543,623 (98.66)
Crash partner driver’s BAC			
Positive Negative	58 (1.00)	5739 (99.00)	5797 (1.05)
	1512 (0.28)	543,686 (99.72)	545,198 (98.95)
Driver’s license status			
No Yes	353 (0.69)	50,661 (99.31)	51,014 (9.26)
	1217 (0.24)	498,764 (99.76)	499,981 (90.74)
Crash partner driver’s license status			
No Yes	109 (0.35)	31,278 (99.65)	31,387 (5.70)
	1461 (0.28)	518,147 (99.72)	519,608 (94.30)
Road speed limit			
≥50 km/h <50 km/h	1209 (0.30)	401,965 (99.70)	403,174 (73.17)
	361 (0.24)	147,460 (99.76)	147,821 (26.83)
Weather conditions			
Adverse Fine	270 (0.27)	98,868 (99.73)	99,138 (17.99)
	1300 (0.29)	450,557 (99.71)	451,857 (82.01)
Light conditions			
Night-time (after sunset) Daytime (after sunrise)	409 (0.29)	139,036 (99.71)	139,445 (25.31)
	1161 (0.28)	419,389 (99.72)	411,550 (74.69)
Distance to nearest trauma centre			
≥5 km <5 km	405 (0.73)	54,983 (99.27)	55,388 (10.05)
	1165 (0.24)	494,442 (99.76)	495,607 (89.95)
Trauma centre level of the nearest trauma centre			
Trauma centre level III Trauma centre level II Trauma centre level I	565 (0.36)	155,825 (99.64)	156,390 (28.38)
	729 (0.28)	256,746 (99.72)	257,475 (46.73)
	276 (0.20)	136,854 (99.80)	137,130 (24.89)
Trauma centre region			
East South Central North	72 (0.53)	13,423 (99.47)	13,495 (2.45)
	693 (0.35)	196,653 (99.65)	197,346 (35.82)
	308 (0.25)	123,740 (99.75)	124,048 (22.51)
	497 (0.23)	215,609 (99.77)	216,106 (39.22)
Vehicle type			
Motorcycle Heavy vehicle Taxi Personal car or van	1496 (0.28)	530,017 (99.72)	531,513 (96.46)
	9 (0.56)	1606 (99.44)	1615 (0.29)
	21 (0.63)	3336 (99.37)	3357 (0.61)
	44 (0.30)	14,466 (99.70)	14,510 (2.63)
Crash partner vehicle type			
Motorcycle Heavy vehicle Taxi Personal car or van	260 (0.11)	240,686 (99.89)	240,946 (43.73)
	355 (1.38)	25,381 (98.62)	25,736 (4.67)
	341 (0.67)	50,485 (99.33)	50,826 (9.22)
	614 (0.26)	232,873 (99.74)	233,487 (42.38)
Crash type			
Head-on Rear-end Sideswipe Other Angle	258 (0.55)	47,021 (99.45)	47,279 (8.58)
	165 (0.25)	67,074 (99.75)	67,239 (12.00)
	172 (0.20)	86,974 (99.80)	87,146 (15.82)
	559 (0.34)	164,754 (99.66)	165,313 (30.00)
	416 (0.23)	183,602 (99.77)	184,018 (33.40)

**Table 3:** Risk factors for fatal injuries among motorcyclists and motorists.

Risk Factors for Fatal Injury	Motorcyclists		Motorists	
	OR	95% CI	OR	95% CI
Driver’s age				
≥65 years 41 to 64 years <18 years 18 to 40 years	6.38	5.63–7.21	1.28	0.56–2.89 0.63–1.66 -
	2.11	1.83–2.40	1.03	
	2.46	1.72–3.52	- *	
	1		1	
Crash partner driver’s age				
≥65 years 41 to 64 years <18 years 18 to 40 years	0.93	0.76–1.14 1.03–1.27 0.51–1.67	0.41	0.10–1.72 0.71–1.80 -
	1.14		1.13	
	0.92		- *	
	1		1	
Driver’s sex				
Male Female	1.78	1.60–1.99	3.93	1.89–8.19
	1		1	
Crash partner driver’s sex				
Male Female	2.34	2.04–2.68	2.34	1.12–4.87
	1		1	
Driver’s use of protective equipment				
Not using a seatbelt or helmet Using a seatbelt or helmet	3.16	2.78–3.58	8.25	5.22–13.05
	1		1	
Driver’s BAC				
Positive Negative	8.30	6.95–9.89	4.31	2.26–8.22
	1		1	
Crash partner driver’s BAC				
Positive Negative	3.95	3.02–5.16	0.91	0.22–3.72
	1		1	
Driver’s license status				
No Yes	2.85 1	2.53–3.22	4.03 1	2.12–7.68
Crash partner driver’s license status				
No Yes	1.23 1	1.01–1.50	1.46 1	0.59–3.62
Road speed limit				
≥50 km/h <50 km/h	1.21 1	1.07–1.36	1.78 1	0.91–3.47
Weather conditions				
Adverse Fine	0.93 1	0.81–1.07	1.16 1	0.68–2.00
Light conditions				
Night-time (after sunset) Daytime (after sunrise)	1.02 1	0.90–1.14	1.43 1	0.90–2.27
Distance to nearest trauma centre				
≥5 km <5 km	3.20 1	2.85–3.60	1.95 1	1.19–3.17
Trauma centre level of the nearest trauma centre				
Trauma centre level III Trauma centre level II Trauma centre level I	1.77	1.52–2.05 1.22–1.62	2.47	1.22–5.01 0.74–3.11
	1.40		1.52	
	1		1	
Trauma centre region				
East South Central North	2.46	1.91–3.16 1.37–1.73 0.95–1.27	0.66	0.16–2.79 0.75–2.15 0.39–1.42
	1.54		1.27	
	1.10		0.75	
	1		1	
Crash partner vehicle type				
Motorcycle Heavy vehicle Taxi Personal car or van	0.40	0.35–0.46 4.53–5.96 2.26–2.97	1.03	0.39–2.77 4.75–14.33 1.07–4.53
	5.20		8.25	
	2.59		2.20	
	1		1	
Crash type				
Head-on Rear-end Sideswipe Other Angle	2.30	1.97–2.70 0.84–1.23 0.72–1.03 1.31–1.70	9.75	4.16–22.85 0.99–4.95 0.54–4.48 0.75–3.88
	1.01		2.22	
	0.86		1.55	
	1.49		1.71	
	1		1	

* Omitted because cell has a value of 0.

**Table 4:** Multiple logistic regression results for motorcyclists and motorists.

Risk Factors for Fatal Injury	Motorcyclists		Motorists	
	OR	95% CI	OR	95% CI
Driver’s age				
≥65 years 41 to 64 years <18 years 18 to 40 years	5.27	4.62–6.01 1.80–2.35 0.80–1.71	1.16	0.50–2.71 0.65–1.76 -
	2.06		1.07	
	1.17		- *	
	1		1	
Crash partner driver’s age				
≥65 years 41 to 64 years <18 years 18 to 40 years	0.77	0.63–0.95 0.74–0.92 0.49–1.77	0.46	0.11–1.97 0.51–1.36 -
	0.82		0.84	
	0.93		- *	
	1		1	
Driver’s sex				
Male Female	1.71 1	1.53–1.92	2.59 1	1.22–5.50
Crash partner driver’s sex				
Male Female	1.48 1	1.28–1.71	0.99 1	0.45–2.21
Driver’s use of protective equipment				
Not using a seatbelt or helmet Using a seatbelt or helmet	2.54 1	2.22–2.90	8.67 1	5.31–14.14
Driver’s BAC				
Positive Negative	4.10 1	3.38–4.98	1.56 1	0.75–3.26
Crash partner driver’s BAC				
Positive Negative	2.61 1	1.97–3.46	0.98 1	0.29–4.17
Driver’s license status				
No Yes	1.69 1	1.48–1.93	2.50 1	1.24–5.04
Crash partner driver’s license status				
No Yes	1.67 1	1.34–2.08	2.08 1	0.79–5.50
Road speed limit				
≥50 km/h <50 km/h	1.29 1	1.14–1.46	2.16 1	1.05–4.44
Weather conditions				
Adverse Fine	0.97 1	0.85–1.12	1.11 1	0.62–1.98
Light conditions				
Night-time (after sunset) Daytime (after sunrise)	1.26 1	1.11–1.43	1.34 1	0.81–1.98
Distance to nearest trauma centre				
≥5 km <5 km	1.98 1	1.75–2.24	1.20 1	0.71–2.04
Trauma centre level of the nearest trauma centre				
Trauma centre level III Trauma centre level II Trauma centre level I	1.30	1.11–1.51 1.03–1.37	1.55	0.74–3.26 0.55–2.40
	1.19		1.14	
	1		1	
Trauma centre region				
East South Central North	1.76	1.35–2.29 1.16–1.49 0.95–1.28	1.01	0.23–4.52 0.76–2.36 0.47–1.79
	1.32		1.34	
	1.10		0.92	
	1		1	
Crash partner vehicle				
Motorcycle Heavy vehicle Taxi Personal car or van	0.43	0.37–0.50 4.01–5.36 1.96–2.59	0.60	0.21–1.73 5.07–17.27 0.91–4.06
	4.63		9.36	
	2.25		1.92	
	1		1	
Crash type				
Head-on Rear-end Sideswipe Other Angle	1.65	1.40–1.95 0.82–1.21 0.59–0.84 1.24–1.61	12.01	4.82–29.88 0.71–3.69 0.41–3.53 0.69–3.66
	0.99		1.62	
	0.70		1.20	
	1.41		1.59	
	1		1	

* Omitted because cell has a value of 0.

**Table 5:** Interaction of distance from the nearest trauma centre with trauma centre region.

Interaction Effect of Hospital Distance and Region ^a^	Motorcyclists		Motorists	
	OR	95% CI	OR	95% CI
≥5 km, eastern region ≥5 km, southern region ≥5 km, central region ≥5 km, northern region <5 km, eastern region <5 km, southern region <5 km, central region <5 km, northern region	5.26	3.69–7.49 2.04–2.92 1.46–2.76 2.06–3.25 0.96–1.99 1.27–1.69 1.01–1.40	1.10	0.13–8.92 0.74–3.25 0.43–3.99 0.38–2.85 0.14–8.11 0.66–2.54 0.39–1.80
	2.44		1.56	
	2.01		1.32	
	2.59		1.04	
	1.38		1.05	
	1.46		1.30	
	1.19		0.83	
	1		1	

^a^ Adjusted for driver’s age, crash partner driver’s age, driver’s sex, crash partner driver’s sex, use of protective equipment, driver’s license status, crash partner driver’s license status, road speed limit, weather conditions, light conditions, trauma centre level, crash partner vehicle type, and crash type.

## Data Availability

The data used for this study is available by request to the authors.
